# High baseline activity in inferior temporal cortex improves neural and behavioral discriminability during visual categorization

**DOI:** 10.3389/fnsys.2014.00218

**Published:** 2014-11-03

**Authors:** Nazli Emadi, Reza Rajimehr, Hossein Esteky

**Affiliations:** ^1^School of Cognitive Sciences, Institute for Research in Fundamental Sciences (IPM)Tehran, Iran; ^2^Research Center for Brain and Cognition, School of Medicine, University of Shahid BeheshtiTehran, Iran; ^3^Howard Hughes Medical Institute and Department of Neurobiology, Stanford University School of MedicineStanford, CA, USA; ^4^McGovern Institute for Brain Research, Massachusetts Institute of TechnologyCambridge, MA, USA

**Keywords:** object recognition, baseline activity, oscillation, decision making, behavioral performance, inferior temporal cortex, macaque monkey

## Abstract

Spontaneous firing is a ubiquitous property of neural activity in the brain. Recent literature suggests that this baseline activity plays a key role in perception. However, it is not known how the baseline activity contributes to neural coding and behavior. Here, by recording from the single neurons in the inferior temporal cortex of monkeys performing a visual categorization task, we thoroughly explored the relationship between baseline activity, the evoked response, and behavior. Specifically we found that a low-frequency (<8 Hz) oscillation in the spike train, prior and phase-locked to the stimulus onset, was correlated with increased gamma power and neuronal baseline activity. This enhancement of the baseline activity was then followed by an increase in the neural selectivity and the response reliability and eventually a higher behavioral performance.

## Introduction

Neurons can spontaneously fire (Wurtz, 1969). This long-known spontaneous or baseline activity in the brain is, by definition, not explicitly associated with a sensory input or a motor output (Ringach, 2009)—though it can show remarkable fluctuations based on the level of attention or expectation when performing a cognitive task (Luck et al., 1997; Kastner et al., 1999; Stokes et al., 2009; van Ede et al., 2010).

The activity induced by an external stimulus interacts with the ongoing baseline activity to evoke the response in the brain (Fox et al., 2006; Liu et al., 2011). fMRI studies have reported that the baseline activity accounts for the trial-to-trial variability in the brain evoked responses (Fox et al., 2006; Becker et al., 2011; Liu et al., 2011). There is also evidence from EEG and modeling studies that the pre-stimulus oscillation interacts with the evoked response (Rajagovindan and Ding, 2011). These findings suggest the existence of a relationship between the baseline activity and the evoked response. However, at the level of single neurons, the underlying mechanisms for such relationship are not clear yet.

The baseline activity has attracted an increasing research interest by the recent findings suggesting that it plays a key role in the perception and behavior (Super et al., 2003). Based on EEG/MEG studies, both power and phase of the “oscillatory” baseline activity are correlated with the perception (Romei et al., 2008; Busch et al., 2009; Addante et al., 2011; Dugue et al., 2011). A large body of fMRI experiments has also shown that the “level” of baseline activity predicts the behavioral performance (Ress et al., 2000; Fox et al., 2007; Hesselmann et al., 2008; Scholvinck et al., 2012). The results of these electrophysiological and neuroimaging studies have changed the traditional view in which the baseline activity was considered noise. However, these human studies do not provide any direct information about the correlation of the baseline activity of single neurons and the behavior. Furthermore, it is not clear how the “oscillation” and the “level” of the neural baseline activity are related to each other. This relationship could integrate numerous earlier electrophysiological (EEG/MEG) and imaging (fMRI) studies that have explored these two phenomena separately.

We addressed these questions by recording from single neurons in inferior temporal (IT) cortex of monkeys performing a visual categorization task. IT neurons show strong selectivity to visual object categories such as faces and bodies (Desimone et al., 1984; Kiani et al., 2007). The activity of these neurons also has a causal influence on the categorical perception, as shown by the microstimulation technique in our previous study (Afraz et al., 2006). The IT cortex, as the final stage of the ventral visual pathway (Logothetis, 1998), is strongly modulated by “top-down” signals involved in attention and expectation (Hochstein and Ahissar, 2002). Since the baseline activity may fluctuate with these top-down feedback signals, the IT cortex can be an ideal choice to investigate the role of baseline activity in the behavior during a complex cognitive task.

## Materials and methods

### Subjects

Two male adult macaque monkeys (*Macaca mulatta*), were used in this study. Monkey 1 and monkey 2 were 9 and 8 years old, respectively. Head restraints and recording chambers were stereotaxically implanted under aseptic conditions on the dorsal surface of the skull of the monkeys while the animals were anesthetized with sodium pentobarbital. All experimental procedures were in accordance with the National Institutes of Health guide for the care and use of laboratory animals. They were also approved by the animal care and use committee of Institute for Research in Fundamental Sciences (IPM).

### Stimuli

The stimuli (7^°^ × 7^°^ in size) were grayscale photographs of bodies (including human, monkey and four-leg) and non-bodies (including planes, cars and chairs). There were 90 images for each category (30 images per subcategory). Each stimulus was presented in four different signal levels. Each signal level was generated by assigning grayscale levels randomly chosen from a uniform distribution to X% of image pixels, where 100-X was the absolute signal level and had one of the values of 90, 70, 55 or 40. These 720 noisy stimuli (180 × 4) and 90 full noise images (0% visual signal) were randomly presented to the monkey, without repetition. Images were repeated across the sessions. In all of the recording sessions (*n* = 61; monkey 1 = 31, monkey 2 = 30) monkeys completed at least half of the trials (minimum = 450, median = 810, mean ± s.e.m. = 791 ± 14). The stimuli were presented on a 19-inch CRT monitor placed 57 cm in front of the monkey seated in a primate chair.

### Task

Monkeys were trained to perform a two-alternative forced-choice body/non-body categorization task. The monkey initiated a trial by fixating on a fixation point within a 2.4° × 2.4° window at the center of the screen for one of the three variable durations (350, 400 or 450 ms). The fixation time was chosen to be variable, to make the situation more similar to the natural environment in which the appearance of a behaviorally relevant sensory stimulus is usually unpredictable. After this fixation period, a noisy image was presented for 70 ms. After a 500-ms blank interval, two small response targets were presented 10° to the left and right of the screen center. The left and right targets represented body and non-body responses, respectively for one monkey and the opposite for the other one. The monkeys were required to make a saccade to the correct target no later than 300 ms after the onset of targets and keep their gaze within 2.4° × 2.4° window on saccade point for 150 ms. The eye position was monitored using an infra-red eye-tracking system. Whenever, the monkey performed the task correctly, a drop of apple juice was delivered into its mouth. For full-noise stimuli (0% visual signal), the monkey was rewarded randomly with a probability of 0.5.

### Recording

Extracellular single-neuron recordings were made on an evenly spaced grid, with 1-mm intervals between penetrations over a wide region of the lower bank of STS, TEp, and TEa cortices (12–18 and 13–20 mm anterior to interauricular line in monkey 1 and monkey 2, respectively). The recording positions were determined stereotaxically by referring to magnetic resonance images acquired before the surgery. Unit responses were recorded through tungsten microelectrodes (FHC Inc.). Spiking activity of 123 visually-responsive single units in IT cortex was recorded from behaving monkeys, during 61 recording sessions (*n* = 49 in monkey 1 and *n* = 74 in monkey 2). Visual responsiveness was defined as significantly larger evoked responses relative to the baseline activity following the presentation of body or non-body images (*t*-test, alpha = 0.05).

### Data analysis

- Based on the similar trend of monkeys' behavior and other results, data from two monkeys were combined in all of the analyses.
- Before running any parametric tests, the normality of the distributions was confirmed by Kolmogorov–Smirnov test (*alpha* = 0.05).
- The variance equality of each two groups that were statistically compared, was confirmed by *F*-test (*alpha* = 0.05).
- All of the *t*-tests were paired *t*-test, unless otherwise mentioned.
- Trials were always aligned to the onset of stimulus presentation, and all of the times mentioned in the manuscript represent the time relative to the stimulus onset.
- For the baseline analysis, −200–0 ms relative to the stimulus onset was used, unless otherwise mentioned. The window used for the analyses of the evoked response was 150–350 ms after the stimulus onset.
- PSTHs were smoothed by convolving with a 15-ms Gaussian kernel.
- For a given neuron, “high baseline trials” (HBT) and “low baseline trials” (LBT) were defined as trials with higher and lower baseline firing rate than the mean baseline activity, respectively.

#### Selectivity index (SI)

The degree of category selectivity of each neuron for body vs. non-body images was measured by SI:

$$SI=\frac{\mu (B)-\mu (O)}{\mu (B)+\mu (O)}\times 100$$

μ(B) and μ(O) were the mean evoked response of each neuron (within 150–350 ms after the stimulus onset) to body and non-body images, respectively. In each neuron, SI values were measured in correct trials of each signal level. Then SI values were averaged across all signal levels. Neurons with SI values larger than zero were considered as “body-selective.”

#### Correct/wrong index (CWI)

As a normalized index of rate modulation between correct and wrong trials, we measured CWI:

$$CWI=\frac{\mu (C)-\mu (W)}{\mu (C)+\mu (W)}\times 100$$

μ(C) and μ(W) were the mean response of each neuron in a specific time window in correct and wrong trials, respectively. In each neuron, the CWI values were averaged across all signal levels. CWI values shown in **Figures 2, 5** were measured in trials were body images were presented.

#### Auto-covariation and fast fourier transform (FFT)

For this analysis, the mean-removed auto-correlation (the auto-covariance) was calculated in each trial of spiking data in 1 ms time bins with this formula:

$$C_{k}=\frac{1}{N-k}\sum_{t\;=\;1}^{N\;-\;k}\left(x_{t}-\bar{x}\right)\left(x_{t\;+\;k}-\bar{x}\right)$$

Where C_*k*_ was an unbiased estimate of the auto-covariance coefficient at lag k. N was the number of points in a time series, and x was the overall mean.

For a given condition (e.g., HBT in body neurons), the auto-covariation functions were then averaged across all trials and all neurons. The amplitude spectrum of the averaged auto-covariation function was obtained using the FFT. We did a permutation test to assess the significance of the deviation of the frequency difference in HBT vs. LBT from chance. Trials of HBT and LBT in each neuron were randomly assigned, while the proportion of each condition was maintained. We calculated the auto-covariation and fast Fourier transform in HBT and LBT for 1000 such permutations. The Amplitude of each frequency in LBT was subtracted from HBT, which resulted in a distribution of amplitude difference. The real difference of amplitudes in our neural population was compared to this distribution. The proportion of the distribution that exceeded the real value was determined as the *P*-value.

#### Time-frequency analysis

We tested whether the spectral power of baseline spiking activity was different between HBT and LBT. In a given neuron, after smoothing the spike train of single trials with a 5-ms Gaussian filter, the power spectrum was obtained using a time-frequency transform (wavelets; EEGLAB software under Matlab) (Dugue et al., 2011). Function of “timefreq” was used with parameters “cycles” and “freqs” set to [1, 5] and [3, 30], respectively. These parameters produce frequencies that increase linearly from 3 to 30 Hz, while the length of the filter increases linearly from 1 to 5 cycles. A 5-ms Gaussian smoothing would have a minimal effect on the frequencies tested here. A 300-ms zero-padding was used to enhance the frequency resolution in our measurements. To plot the mean power spectrum in each condition, after averaging the power spectrum of the trials of one neuron, the mean was measured across the population of body neurons. The difference of the power spectrum in HBT vs. LBT was measured by subtracting the averaged power spectrum of the population of body neurons in LBT from the same in HBT. The significancy of this difference was tested using a permutation test. The power spectrum was measured after the association between trials and conditions (HBT or LBT) in each neuron was randomly assigned, while keeping the number of trials in each condition constant. A distribution of the spectral power difference was obtained after 1000 such permutations. The experimentally observed difference in the power spectrum of HBT vs. LBT was compared to this distribution to evaluate its deviation from chance. The proportion of the randomized generated values that exceeded the experimentally obtained difference was determined as the *P*-value (Dugue et al., 2011).

#### Phase locking

We tested the difference of the phase locking between HBT and LBT. In each neuron, phase information in HBT and LBT conditions was obtained using the time-frequency transform, as described in the time-frequency analysis. The level of phase concentration across trials was quantified by the phase-locking factor (PLF) or inter-trial coherency (ITC) (Busch et al., 2009). PLF is a measure of event-related phase consistency of neuronal responses and was measured with this formula:

$$PLF=\frac{1}{n}\left(\sqrt{{\left(\sum_{i\;=\;1}^{n}\cos\theta_{i}\right)}^{2}+{\left(\sum_{i\;=\;1}^{n}\sin\theta_{i}\right)}^{2}}\right)$$

Where n is the number of trials and θ_*i*_ is the phase angle at the ith trial. PLF takes values between 0 and 1, representing the amount of synchronization across trials between the spike train and a specific event. For this analysis, the amplitude of each frequency in single trials was measured during −300–100 ms relative to the stimulus onset (**Figures 4A,B**). In each neuron, the oscillatory trials with amplitude above the 25th percentile were selected in HBTs and LBTs for the phase analysis. PLF was measured at each frequency and time point. By subtracting the phase locking values in LBT from HBT, we calculated the difference of phase locking between these two conditions. Our results did not depend on the selected window or the percentile. We did a permutation test to assess the significance of the deviation of the PLF in HBT vs. LBT from chance. Phases of high and low baseline trials in each frequency and time bin were randomly assigned, while the proportion of each condition was maintained. We calculated the phase locking difference in HBT vs. LBT for 1000 such permutations. The real phase locking difference in our data was compared to this distribution. The proportion of the randomized generated distribution that exceeded the data-driven difference was determined as the *P*-value.

#### Coupling of theta oscillation with spike probability and gamma power

First, we performed the time-frequency analysis and the phase calculation for the theta rhythm (3–7 Hz), as described above. Here we included the peri-stimulus period of all the trials (HBT and LBT) in the analysis. The theta troughs were identified as a time-point where the phase value was larger than the phase value of its following time-point by more than 5 radians (287 degrees or 1.6π) (Canolty et al., 2006). 200-ms epochs, centered on the time-points of theta troughs, were extracted from the spike trains, and the mean spike probability was calculated. To measure gamma power, after smoothing the spike train of single trials with a 5-ms Gaussian filter, the power spectrum was obtained using a time-frequency transform (wavelets; EEGLAB software under Matlab) (Dugue et al., 2011). Function of “timefreq” was used with parameters “cycles” and “freqs” set to [5, 15] and [30, 90], respectively. These parameters produce frequencies that increase linearly from 30 to 90 Hz, while the length of the filter increases linearly from 5 to 15 cycles. To explore the coupling between the theta rhythm and the gamma power, 300-ms epochs centered on the time-points of theta troughs were extracted from the power spectrum of gamma rhythm, and the mean gamma power was calculated.

#### Fano factor (FF)

Fano factor was used as an index of response variability:

$$FF=\frac{\sigma^{2}}{\mu }$$

σ2 and μ were the variance and mean of the spike count, respectively. The FF was measured in 100-ms windows with 1-ms steps. First and last windows were centered at 150 ms and 350 ms after the stimulus onset. The number of spikes was calculated in each window in each trial. Then the FF within each window was computed as the ratio of variance in spike counts to mean spike count across all trials. For rate matching among HBT and LBT conditions in each neuron, the most common firing rate across all windows in both conditions was selected as the matching rate. The FFs of all windows with firing rates ±10% of the selected rate were averaged in each condition. At least 10 such windows were needed for each neuron to be included in the analysis. 65 body neurons met these criteria and were included in the FF analysis.

#### Classifier

A linear “support vector machine” was used to assess the neural performance. For a given condition (e.g., HBT in body neurons), the evoked response of neurons (within 150–350 ms after the stimulus onset) to body and non-body images across signal levels was used as an input to the classifier. In each round of classification, we randomly selected the 75% of trials from every neuron for training the classifier. The classification performance of the neural population was tested on the remaining 25% of trials. In all of the neurons, equal numbers of HBT and LBT trials were pseudorandomly selected from the pool of trials in each condition. This procedure was repeated for 1000 rounds to evaluate the statistical difference in performance between conditions.

#### Behavioral dprime

To assess monkeys' behavioral performance, we calculated the behavioral dprime:

dprime=Z(hit)−Z(false alarm)

Z(hit) and Z(false alarm) were the z-transforms of “hit rate” and “false alarm,” respectively. “Hits” and “false alarms” were trials that the monkey categorized the presented image as body correctly and incorrectly, respectively.

## Results

### Behavioral task

**Figure 1:**
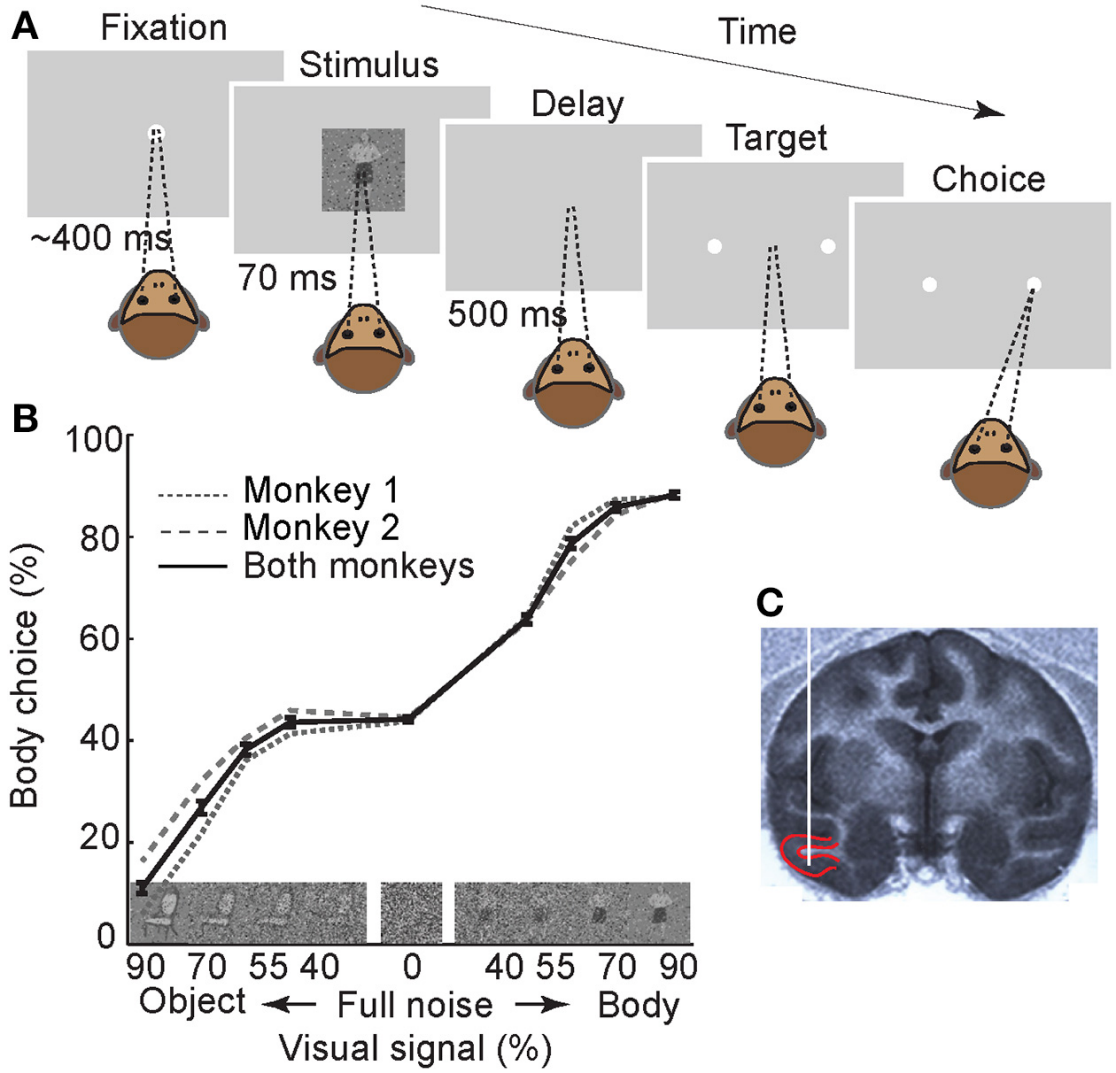
**Experimental paradigm and behavioral results**. **(A)** Monkeys were trained to perform a two-alternative forced-choice body/non-body categorization task. The stimulus set contained 720 photographs of body and non-body images, in four different signal levels, and 90 full-noise stimuli. For illustration only, stimulus is depicted here with a relatively large size compared to the monitor screen. Numbers represent the duration of each epoch. **(B)** Monkeys' performance. Images on the X-axis are examples of noisy stimuli in different signal levels. The error bars represent the ±1 standard error of the mean (s.e.m.) across recording sessions (*n* = 61). **(C)** Sagittal section of the MRI at the anteroposterior level of 16 in monkey 1. Red lines depict the boundaries of recording area (lower bank of STS and TE). White vertical line schematically represents the inserted electrode.

Two macaque monkeys were trained to perform a two-alternative forced-choice “body/non-body” categorization task (Figure 1A). In this task, monkeys initiated the trials by fixating a central fixation point for ~400 ms. This pre-stimulus phase was followed by a brief (70 ms) presentation of an image. The image was chosen pseudo-randomly from a set of pictures of body or non-body images that were degraded by noise (Emadi and Esteky, 2013). We used different noise levels to create a range of task difficulties and to obtain psychometric functions. After a delay of 500 ms, two targets (saccade points) were presented, and the monkey was required to make a saccadic eye movement to one of the targets to indicate whether the image was a body or a non-body. Each correct response (i.e., when the image was correctly categorized by monkey) was rewarded by a drop of juice. For “full-noise” stimuli (0% visual signal), the monkey was rewarded randomly in 50% of the trials. These trials were excluded during the analysis of neural data. The performance of monkeys in this categorization task was plotted as the percentage of “body” choices for the various noisy stimuli (Figure 1B). As expected, monkeys had a better performance when categorizing less noisy stimuli. There was a minimal bias toward non-body choices in both monkeys (performance at full noise, mean ± s.e.m.: monkey 1 = 47.6 ± 0.6, monkey 2 = 48.3 ± 0.8). We observed better performance for body compared to non-body images at 40% signal level in both monkeys. This could be attributed to the smaller effect of the high-frequency noise on image contours used in body detection (Downing et al., 2001). It can also be due to a higher noise tolerance in detecting bodies as a behaviorally important category (Downing et al., 2006; Pitcher et al., 2012).

We recorded the spiking activity of 123 visually-responsive single neurons in IT cortex (Figure 1C) (*n* = 49 in monkey 1 and *n* = 74 in monkey 2). Using a selectivity index that indicates how selectively the neurons responded to body images vs. non-body images (see Experimental Procedures), 75 neurons were classified as “body-selective” and were further analyzed (*n* = 35 in monkey 1 and *n* = 40 in monkey 2). These body neurons were all excited by body presentation and not inhibited.

### Baseline firing rate and behavior

**Figure 2:**
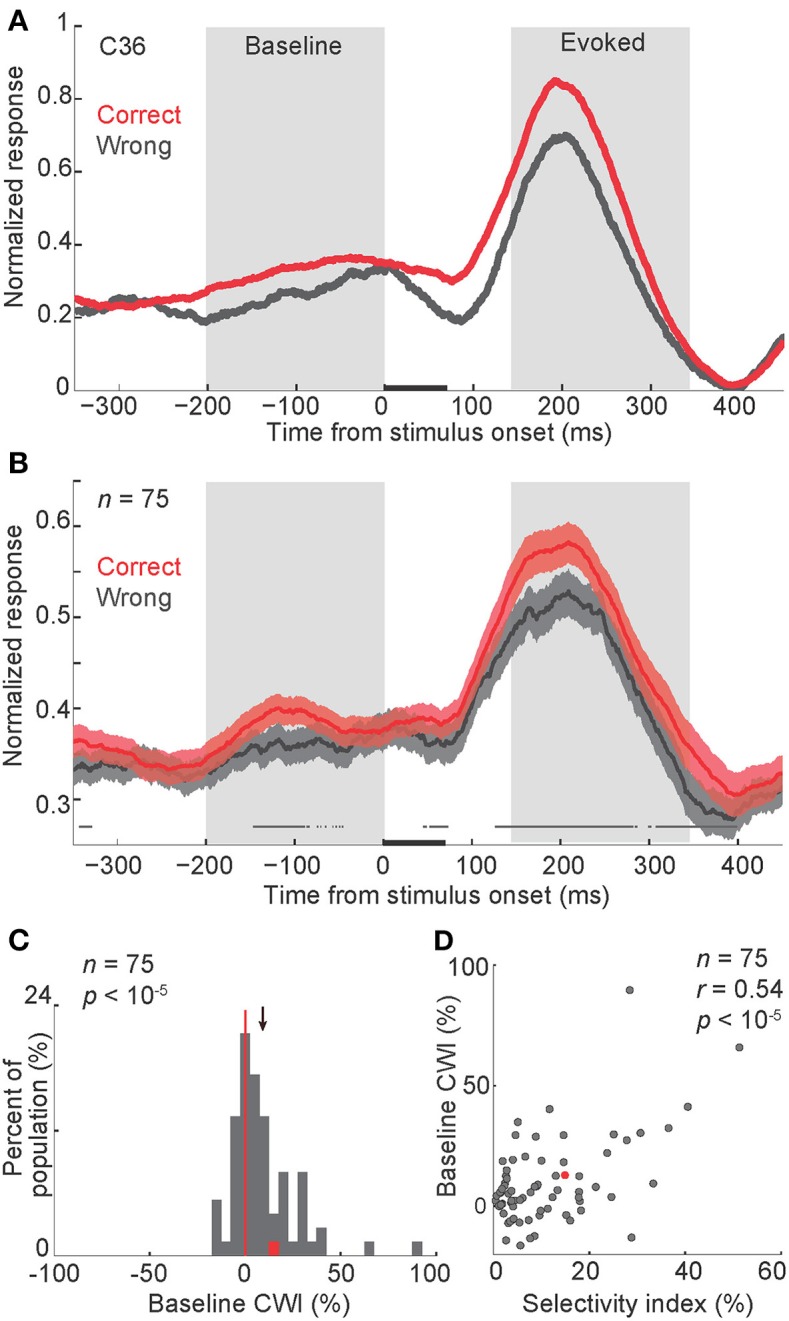
**Modulation of baseline activity in correct vs. wrong trials**. **(A,B)** Normalized firing rate in correct and wrong trials, in a representative body neuron (C36, selectivity index = 0.15) **(A)**, and across all neurons **(B)**. In each neuron and each signal level, the peak response was measured separately in correct and wrong trials. The larger peak was selected to normalize both correct and wrong trials. Finally the normalized firing rates were averaged across noise levels. The gray boxes represent periods of baseline and evoked activity used for the further analysis. In **(B)** shaded areas represent s.e.m. of correct and wrong responses across the population. The line above the X-axis represents the significant difference between correct and wrong responses, obtained by paired *t*-test in 50-ms sliding windows with 1-ms steps, plotted at the middle of each bin (*t*-test, *alpha* = 0.05). Stimuli were presented for 70 ms, represented by a black bar on the X-axis. *P*-values represent the *t*-test results for comparing the firing rates between correct and wrong trials during baseline or evoked windows. “n” here and in all other figures represents the number of body neurons included in the analysis. **(C)** Histogram of the CWI (correct/wrong index) in the baseline period. The red data-point corresponds to the representative neuron shown in panel a (C36). The red vertical line shows the line of no difference (zero). The arrow represents the mean of the distribution. **(D)** The relationship of baseline CWI with the selectivity index. Each data-point corresponds to one neuron. Conventions as described in **(C)**.

To evaluate the relationship between the baseline activity and the behavioral performance, we plotted the response of body neurons to their preferred category (body images) in “correct” and “wrong” conditions (Figures 2A,B). A 200-ms window just before the stimulus onset was used for further analyses of the baseline activity. The averaged firing rate in this window was 7.2 ± 0.7 Hz. The modulation of baseline firing rate in correct vs. wrong trials, as defined by a “correct/wrong index” (abbreviated as CWI, see Experimental Procedures), was calculated in each body neuron. For the population of neurons, this index was significantly positive, meaning that baseline firing rate was higher in correct compared to wrong trials (Figure 2C, CWI = 9.2 ± 2% [mean ± s.e.m.], *t*-test, *P* < 10^−5^). We also found that baseline CWI was correlated with the selectivity index of neurons (Figure 2D, Pearson correlation, *r* = 0.54, *P* < 10^−5^). This result suggests that the relationship between the baseline firing rate and the behavior was correlated with the category selectivity of body neurons. Including both correct and wrong trials to measure selectivity index did not change the results of Figures 2C,D.

### Baseline spiking and oscillatory activity

**Figure 3:**
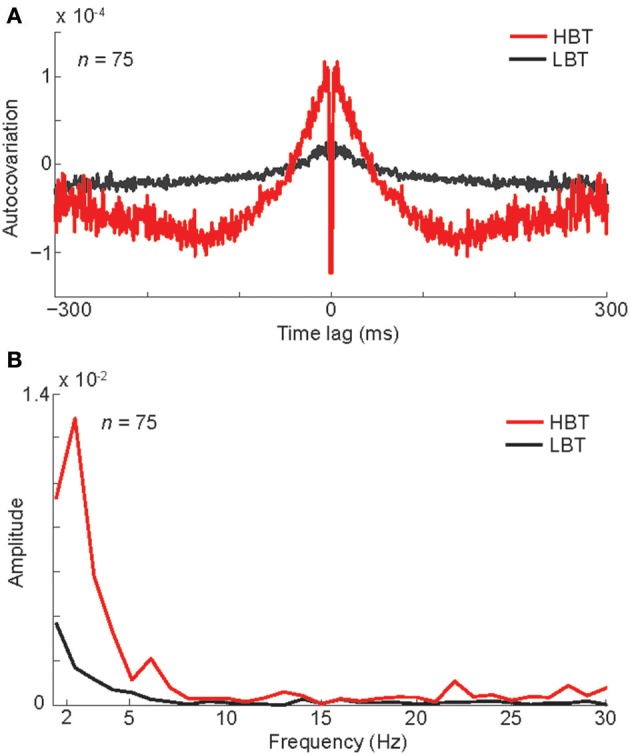
**Oscillation associated with different levels of baseline activity**. **(A)** The auto-covariation plot for high baseline trials (HBT) and low baseline trials (LBT), measured during the baseline period (−300–0 ms). Low auto-covariation within 2 ms of the central time bin reflects the absolute refractory period of the isolated single units. **(B)** The FFT amplitude of the auto-covariation in HBT and LBT.

Next we asked whether this baseline rate modulation was part of an ongoing oscillation in the neural spiking activity. To evaluate the relationship between the baseline firing rate and the underlying oscillatory activity, we measured the auto-covariation function separately in “high baseline trials” (HBT) and “low baseline trials” (LBT) (see Experimental Procedures). For a given neuron, HBT and LBT were defined as trials with higher and lower baseline firing rate than the mean baseline activity, respectively. The averaged auto-covariation function of the baseline activity demonstrated a low-frequency oscillation in HBT (Figure 3A). This oscillation was virtually absent in LBT (Figure 3A). To quantify this oscillatory activity, we did a fast Fourier transform (FFT) on the auto-covariation functions of baseline activity (Figure 3B). This analysis showed a larger amplitude in the low-frequency rhythms, in HBT compared to LBT. To statistically confirm this finding, we used a permutation test and found significantly larger amplitude in 2–7 Hz, in HBT compared to LBT (permutation test, *P* < 0.001). This frequency range overlaps with the delta and theta bands. Since our monkeys were not cued about the upcoming stimulus, such oscillatory activity was also expected to happen prior to the presentation of stimuli from the non-preferred category. Our analyses confirmed the presence of a low-frequency oscillation in HBT, in trials in which non-preferred stimuli were presented. Correspondingly the FFT results revealed significantly larger amplitude in 2–7 Hz, in HBT compared to LBT, in those trials (permutation test, *P* < 0.001). These findings suggest a relationship between the firing rate and the low-frequency (<8 Hz) oscillation of baseline activity.

**Figure 4:**
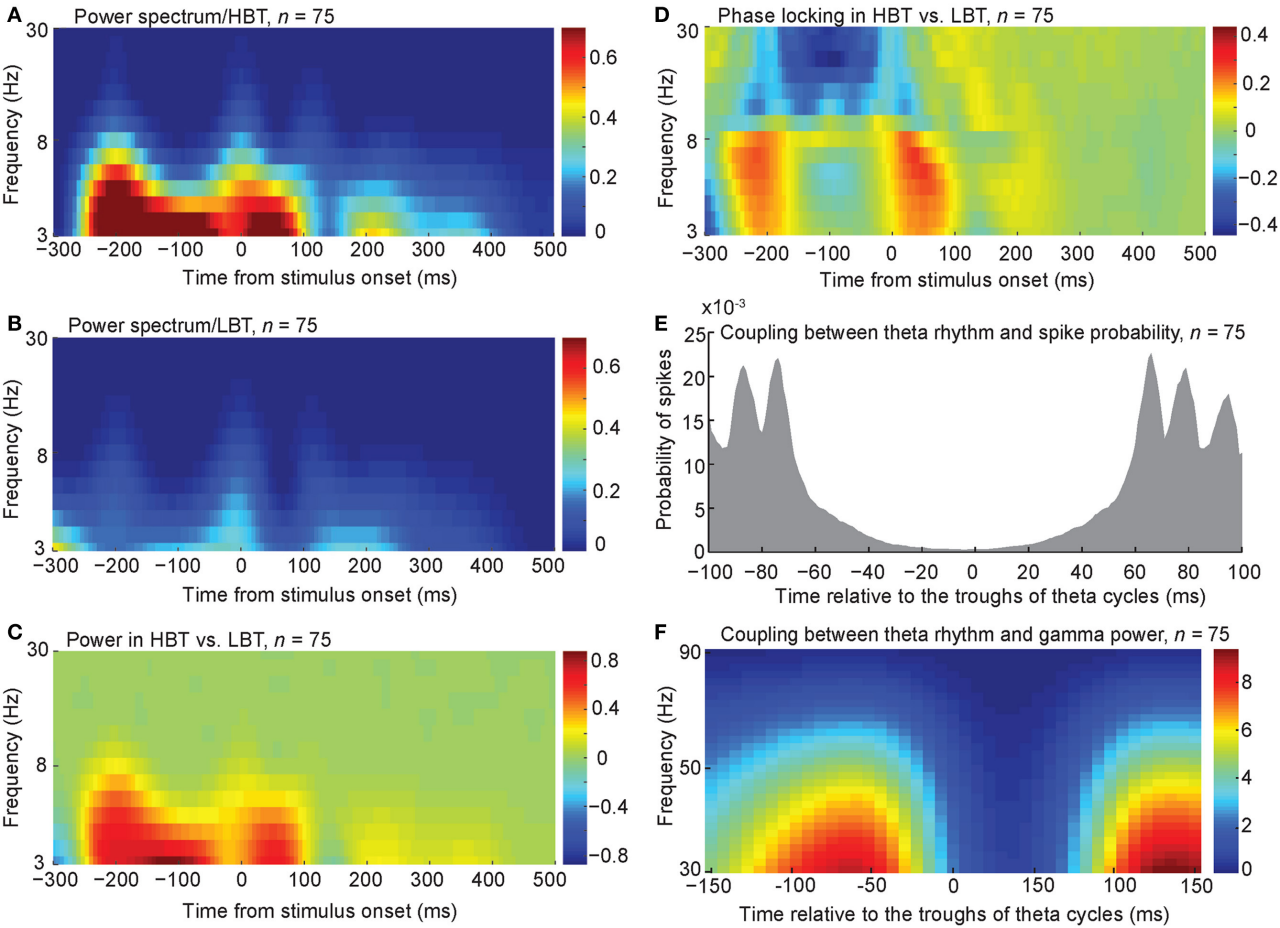
**Spectral power and phase associated with different levels of baseline activity**. **(A,B)** The averaged spectral power of the population of body neurons in HBT **(A)**, and LBT **(B)**. **(C)** The difference of the spectral power in HBT vs. LBT. **(D)** The difference of the phase locking in HBT compared to LBT. **(E)** Coupling between the theta troughs and the spike probability. **(F)** Coupling between the theta troughs and the gamma power.

To further explore the baseline oscillatory activity at each frequency and time point, we performed a time-frequency analysis on the spike trains of HBT and LBT (see Experimental Procedures). Consistent with the auto-covariation results, the power of low-frequency oscillation (<8 Hz) was larger during the baseline period, in HBT compared to LBT (Figures 4A,B,C, permutation test, *P* < 0.001). This difference disappeared around the onset time of the evoked response [~100 ms after the stimulus onset, corresponding to the latency of the evoked responses in the IT cortex (Kiani et al., 2005)].

To test the level of synchronization at different frequencies in HBT vs. LBT, we calculated the phase locking factor (PLF, see Experimental Procedures). PLF is a measure of event-related phase consistency of neuronal responses across trials (Tallon-Baudry et al., 1997; Delorme and Makeig, 2004; Roach and Mathalon, 2008; Busch et al., 2009). We found that, during the baseline period (~200 ms before the stimulus onset), PLF in the low-frequency rhythms (<8 Hz) was larger in HBT compared to LBT (Figure 4D, permutation test, *P* < 0.001). This difference was also evident just before the evoked response. We also found a desynchronization in the alpha and beta rhythms (9–30 Hz) during the baseline period. This can be related to the previously reported desynchronizing effects of attention on these frequency bands (Capotosto et al., 2009; van Ede et al., 2011).

The relationship between the observed low-frequency oscillation and the spike probability was examined by aligning the spikes relative to the troughs of the theta-frequency oscillation. We included all trials (HBT and LBT) in this analysis. We found a coupling between theta troughs and spike probability during the baseline period (Figure 4E). This coupling was consistent with the higher power of low-frequency rhythms in HBT vs. LBT shown in Figure 4C. We observed several distinct peaks in the spike probability plot, which could be an indication of cross-frequency coupling between theta and gamma rhythms. We tested this possibility by calculating the gamma power relative to the troughs of the theta-frequency oscillation. This analysis revealed a cross-frequency coupling between theta and low gamma (<50 Hz) bands (Figure 4F). By performing the FFT analysis on the auto-covariation functions of baseline activity, we also found a larger amplitude in the gamma band, in HBT compared to LBT (permutation test, *P* < 0.001). We further performed a time-frequency analysis on the spike trains of HBT and LBT in the gamma band (30–90 Hz). Consistent with the auto-covariation results, the power of gamma oscillation was larger during the baseline period, in HBT compared to LBT (permutation test, *P* < 0.001).

### Correlation of baseline and evoked responses

**Figure 5:**
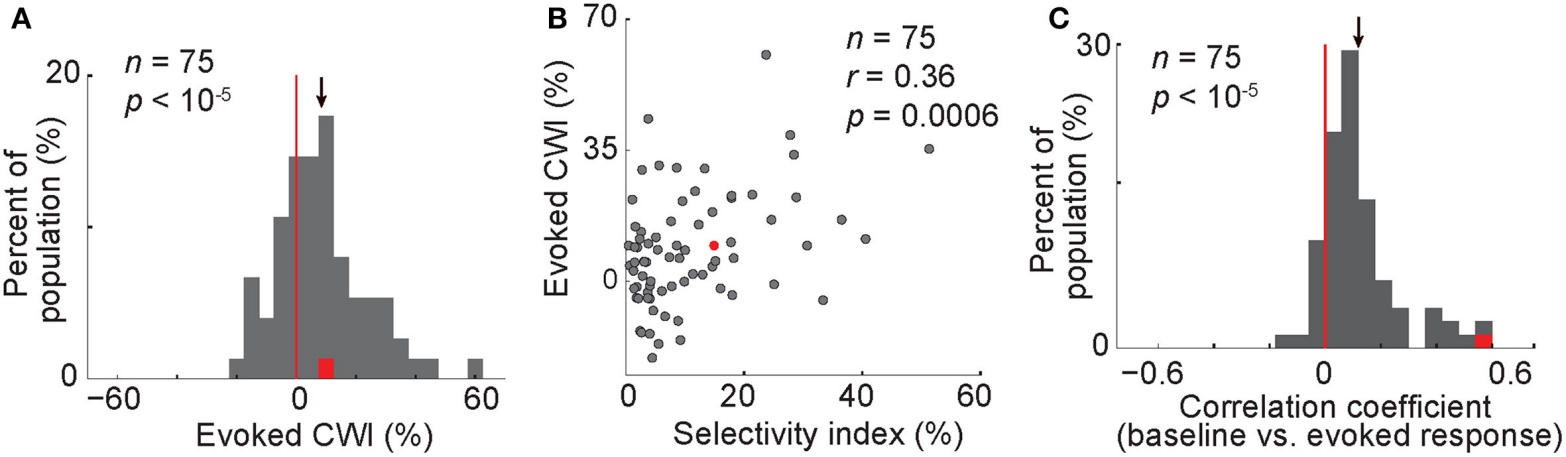
**Modulation of the evoked response in correct vs. wrong trials; correlation of baseline and evoked responses**. **(A)** Histogram of the CWI of the evoked response. **(B)** The relationship of the evoked CWI with the selectivity index. **(C)** The histogram representing the correlation of baseline and evoked firing rate. Each data point shows the correlation coefficient value for one body neuron. Conventions as described in Figure 2.

So far our findings suggest that in trials with a synchronized low-frequency (<8 Hz) oscillation, a higher number of spikes occur before the stimulus onset. We also showed that a higher baseline activity was correlated with an improved behavioral performance. Since the behavioral choice is immediately preceded by the evoked response, we asked whether the evoked firing rate was also correlated with the behavior. Figure 2B shows a difference between the evoked firing rate in correct and wrong trials. A 200-ms window from 150 to 350 ms after the stimulus onset was selected for the further analyses of the evoked response (rate_c_ > rate_w_ in this time window, *t*-test, *P* = 0.00009). The CWI was measured in this window of the evoked response, in each body neuron. For the population of neurons, this index was significantly positive, meaning that the evoked firing rate was higher in correct trials compared to wrong trials (Figure 5A, CWI = 8.4 ± 1.7%, *t*-test, *P* < 10^−5^). The evoked CWI was not significantly different from the baseline CWI in the population of body neurons (*t*-test, *P* = 0.37). We also found a positive correlation between the evoked CWI and the selectivity index of neurons (Figure 5B, Pearson correlation, *r* = 0.36, *P* = 0.0006). Including both correct and wrong trials to measure selectivity index did not change the results of Figures 5A,B. Thus, similar to the baseline activity, the relationship between the evoked response and the behavior was correlated with the category selectivity of body neurons. This similarity suggests that the baseline activity and the evoked response may be correlated on a trial-by-trial basis. To confirm this, we measured the correlation between baseline and evoked firing rates in each neuron, across trials. We found significant positive correlation in 44% of body neurons (Pearson correlation, *r* > 0, *P* < 0.05). We also observed a correlation between baseline and evoked responses across the population with an average correlation coefficient of 0.124 ± 0.0173 that was significantly larger than zero (Figure 5C, *t*-test, *P* < 10^−5^). The correlation between the baseline activity and the responses evoked by the non-preferred category was also positive and significant (average *r* = 0.123 ± 0.0174, *t*-test, *P* < 10^−5^). All these results suggest that trials with higher baseline activity also show higher evoked responses.

### Relationship between baseline activity and selectivity of the evoked response

**Figure 6:**
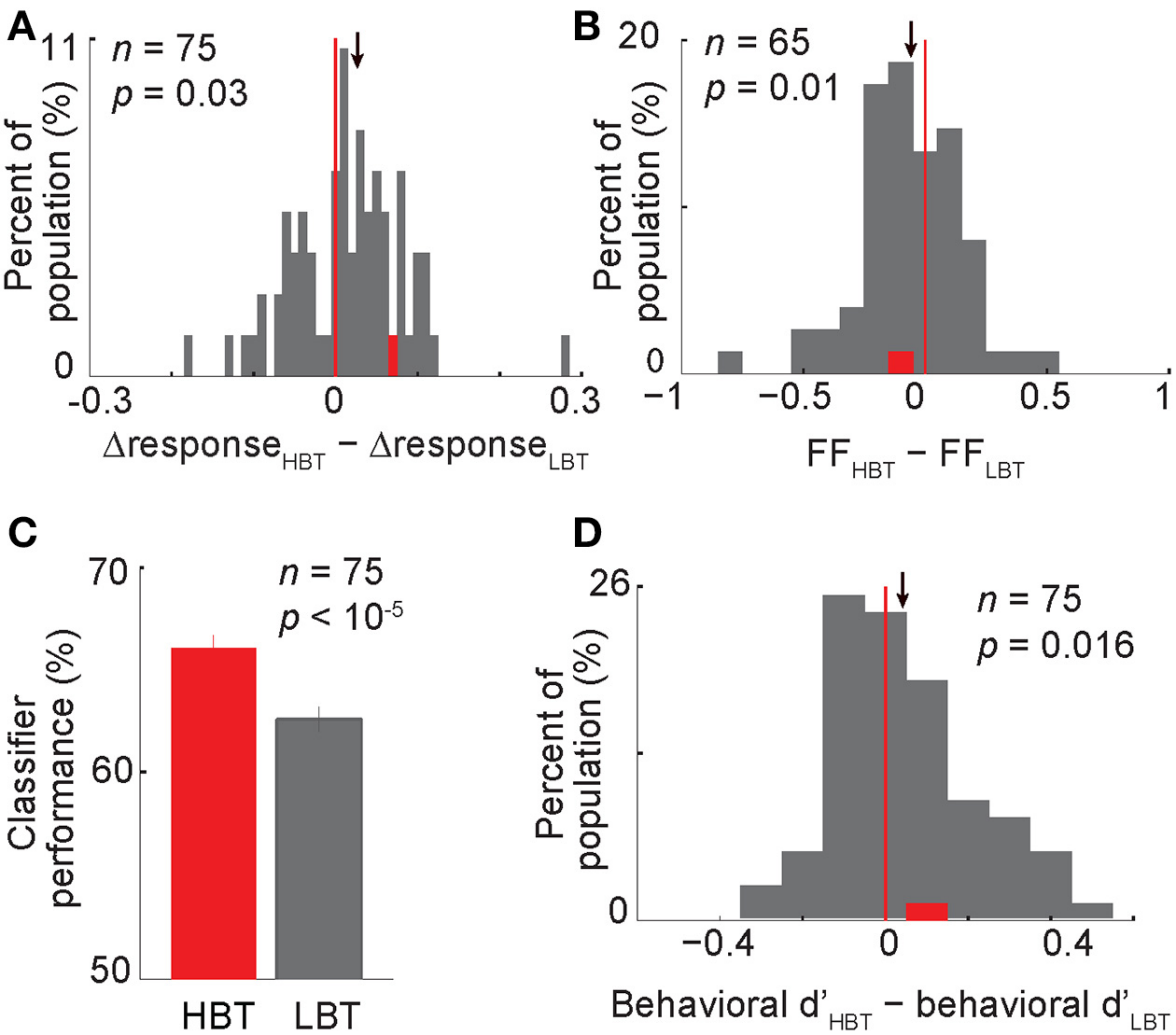
**Contribution of the baseline activity to the evoked response, neural and behavioral performance**. **(A)** The histogram showing the modulation of the differential neural responses in HBT vs. LBT. This differential response (?response) was obtained by subtracting the normalized evoked response to non-body images from the normalized evoked response to body images. One data point (*X* = 0.91) was larger than the X-axis limit and is not shown here **(B)** The modulation of the rate-matched Fano factor in HBT vs. LBT. **(C)** The performance of a neural classifier in HBT and LBT. Error bars indicate the s.e.m. over 1000 repetitions of the classification in each condition. **(D)** The modulation of the behavioral dprime (d′) in HBT vs. LBT. Conventions as described in Figure 2.

Based on the observed correlation between the baseline activity and the evoked response, we hypothesized that the baseline activity interacts with the evoked response to influence the neural coding and eventually the behavioral performance. This idea was supported when we compared different aspects of the evoked responses in HBT and LBT. We first measured the “neural discriminability” (as defined by the differential response to body vs. non-body images) in HBT and LBT. This metric was significantly higher in HBT than LBT across the population (Figure 6A, Δresponse_HBT_ − Δresponse_LBT_ = 0.027 ± 0.014, *t*-test, *P* = 0.03). This finding suggests that despite a rate enhancement in HBT for both preferred and non-preferred categories (positive correlation of baseline and evoked firing rates for both categories), the magnitude of this effect was still larger for the preferred category. This could result in better discrimination of preferred vs. non-preferred categories. The enhanced neural discriminability in the evoked responses of HBT suggests a multiplicative response gain modulation in these trials (McAdams and Maunsell, 1999; Treue and Martinez Trujillo, 1999; VanRullen et al., 2006).

### Relationship between baseline activity and variability of the evoked response

In addition to the response amplitude for preferred vs. non-preferred categories, the variability of the evoked response within each category could also affect the category selectivity of neurons. It has been reported that visual attention decreases the variability of the stimulus-evoked response (Mitchell et al., 2007). However, the relationship between the modulation of baseline activity and the variability of evoked response is not known. To address this issue, we measured the response variability using rate-matched Fano factor (FF, see Experimental Procedures). Interestingly, we found that the rate-matched FF was lower in HBT than LBT, for the preferred category (Figure 6B, ΔFF FF_HBT_ − FF_LBT_ = −0.06 ± 0.02, *t*-test, *P* = 0.01). A similar trend was also observed for the non-preferred category (ΔFF FF_HBT_–FF_LBT_ = −0.04 ± 0.029, *t*-test, *P* = 0.06). Lower response variability for both preferred and non-preferred categories could enhance the differentiation between the two response distributions. Thus, unlike the firing rate which was selectively modulated, the response reliability was increased independent of which category was presented. This suggests the possibility of two different mechanisms, one selectively affecting the response amplitude and the other non-selectively changing the response variability, both acting to maximize the category selectivity of neurons.

### Relationship between baseline activity and neural/behavioral performance

Given an enhancement in neural discriminability and response reliability in HBT, we predicted an improved neural and behavioral performance in HBT compared to LBT. The neural performance, assessed by a linear classifier trained on the population of neurons to discriminate body and non-body images (see Experimental Procedures), was higher in HBT [Figure 6C; Δperformance (performance_HBT_ − performance_LBT_) = 3.5 ± 0.6%, *P* < 10^−5^]. The behavioral performance, quantified by dprime index (see Experimental Procedures), was also significantly higher in HBT [Figure 6D; Δd′ (d′_HBT_ − d′_LBT_) = 0.04 ± 0.02, *t*-test, *P* = 0.016]. All these results suggest that an improved response selectivity and reliability, which happened in trials with a stronger low-frequency oscillation and a higher baseline firing rate, has resulted in more efficient neural coding and better behavioral performance.

**Figure 7:**
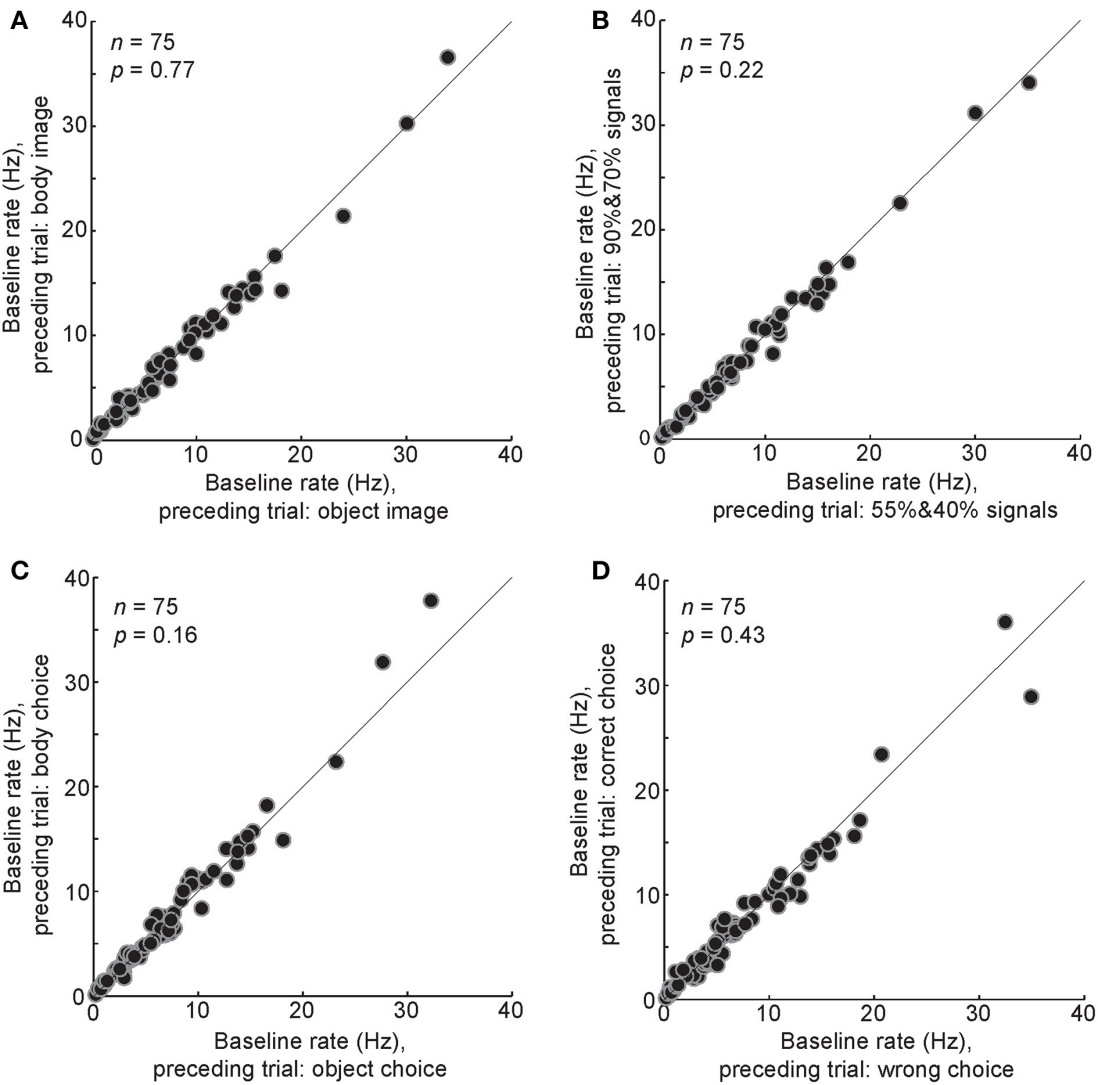
**Relationship between different events in the preceding trial and the baseline airing rate in the following trial**. The baseline firing rates were compared between different conditions of the last trial: a body or a non-body image was presented **(A)**, a high-noise (90 and 70%) or low-noise (55 and 40%) image was presented **(B)**, a body or a non-body choice was made by the monkey **(C)**, a correct or a wrong choice was made by the monkey **(D)**. Each data point shows the one body neuron.

The baseline activity in a given trial could be potentially modulated by various events in a preceding trial. To test this, trials were divided into two groups based on a particular event in the preceding trial, then the baseline firing rates in these two groups were compared. We did this analysis for the following conditions: (1) the image in the preceding trial was a body or a non-body (Figure 7A, *t*-test, *P* = 0.7), (2) the image in the preceding trial was a low-noise image (90 or 70% noise levels) or a high-noise image (55 or 40% noise levels) (Figure 7B, *t*-test, *P* = 0.2), (3) the monkey's choice in the preceding trial was a body choice or a non-body choice (Figure 7C, *t*-test, *P* = 0.16), (4) the monkey's response in the preceding trial was a correct response (“reward” condition) or a wrong response (“no reward” condition) (Figure 7D, *t*-test, *P* = 0.4). The results showed no significant modulation of the baseline firing rate by different events in the preceding trial.

**Figure 8:**
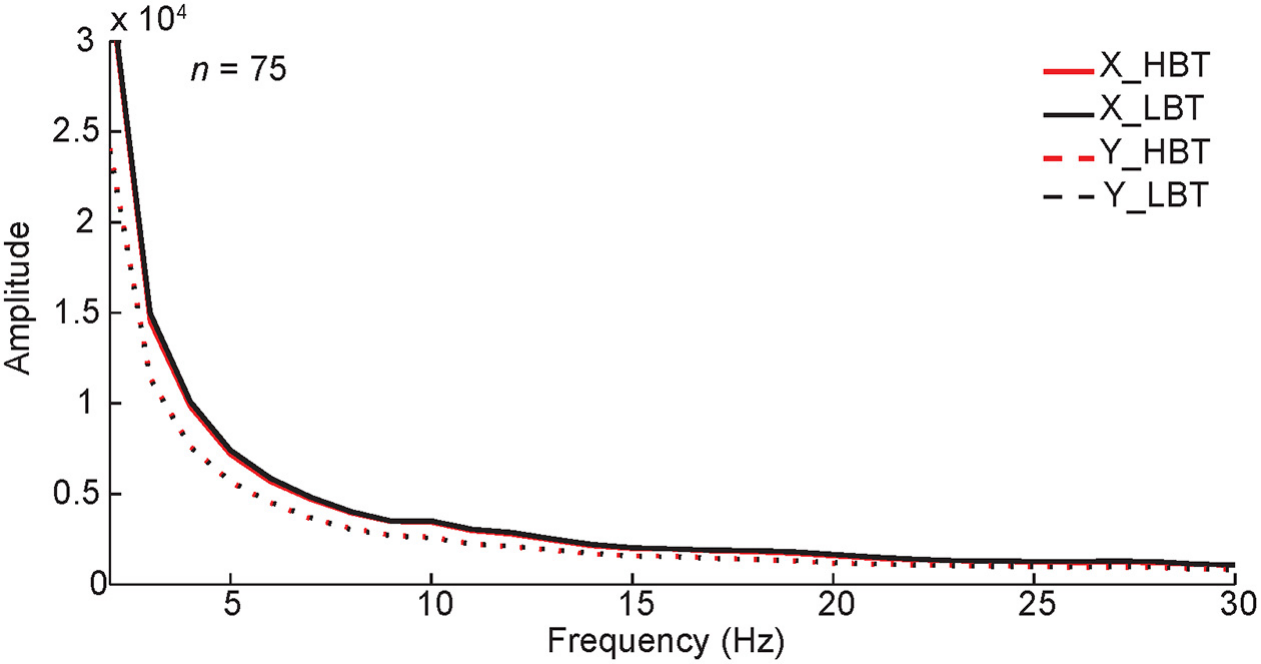
**Monkeys' eye movements in HBT and LBT**. The power spectrum of the monkeys' horizontal (X) and vertical (Y) eye positions in single trials, during baseline period (−300–0 ms), is shown. To test if the results were different between HBT and LBT we performed a permutation test, separately for horizontal and vertical positions. In the permutation test, the trials were randomly assigned to HBT and LBT while keeping the number of trials in each condition unchanged. We compared the experimental spectral power difference with the distribution of spectral power differences obtained from 1000 such permutations. The results showed that the difference between the spectral power of HBT vs. the spectral power of LBT, for the horizontal or vertical positions, was not significant (*P* > 0.05).

During the baseline period, monkeys maintained their fixation on the fixation point, within a fixation window. However, small eye movements (microsaccades) within that window were possible. We explored the spectral pattern of eye movements during the baseline period and compared in HBT vs. LBT to rule out any potential effects on our results. For this, the power spectrum of horizontal (X) and vertical (Y) eye positions was computed for the baseline period, in HBT and LBT (Figure 8). There was no difference in the power spectra of eye data between HBT and LBT (permutation test, *P* > 0.05). The incidence of small eye movements was also measured for the baseline period, in HBT and LBT (HBT = 1.29 ± 0.59, LBT = 1.33 ± 0.50). Again we found no difference in the number of small eye movements between HBT and LBT (*t*-test, *P* = 0.67).

## Discussion

By recording from the single neurons in the IT cortex of monkeys performing a visual categorization task, we found a chain of neural events that linked the baseline activity to cortical sensory processing and perception: (1) emergence of oscillatory activity in a low-frequency range (<8 Hz) during the baseline period, (2) phase locking of this oscillation to the stimulus onset, (3) coupling of low- and high-frequency rhythms, which was accompanied by a “baseline shift” in a critical time window just before the stimulus onset, (4) consequent improvement in the selectivity and reliability of neuronal evoked responses, and (5) correct behavioral choice.

**Figure 9:**
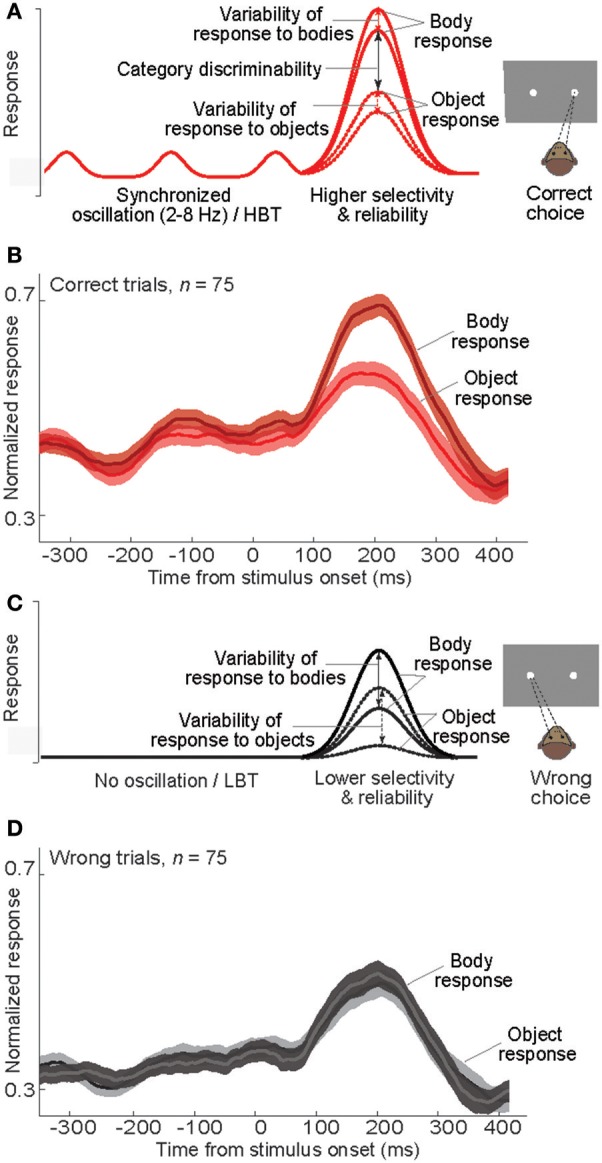
**Neural events following baseline modulation during a categorization task**. **(A)** Schematic diagram of the mean response of a model body neuron responding to body and non-body images in “correct” trials. The impact of rhythmic baseline modulation on the neural response and behavior is illustrated. X-axis as in **(B)**. **(B)** Plot of normalized averaged firing rate of body neurons in correct trials. In each neuron and each signal level, the firing rates were normalized by the peak response, and then the normalized firing rates were averaged. **(C)** Schematic diagram of the mean response of a model body neuron responding to body and non-body images in “wrong” trials. The impact of no baseline activity on the neural response and behavior is illustrated. X-axis as in **(D)**. **(D)** Plot of normalized averaged firing rate of body neurons in wrong trials. Normalization was done similar to **(B)**.

Using a schematic model, we have summarized our findings to describe how a correct choice is made during the categorization task (Figure 9). A synchronous oscillation of baseline activity occurs across a population of IT neurons. The strength of such synchronous oscillatory activity can vary across trials, based on the level of cognitive factors such as attention and motivation. In trials with a strong low-frequency (<8 Hz) oscillation, that is coupled with the gamma band and phase-locked to the stimulus onset, a peak of the oscillation can effectively occur before the stimulus presentation; a situation that results in an apparent “baseline shift.” Another peak of the oscillation can also occur around the stimulus presentation, which would enhance the neural responsiveness and produce an elevated evoked response. The enhanced baseline and evoked activity in these oscillatory/HBT trials subsequently increases neural selectivity and reduces response variability (two signatures of an improved neural performance (Treue and Martinez Trujillo, 1999; Mitchell et al., 2007). A higher neural performance would eventually lead to an increased probability of correct choices (Figure 9A). Consistent with this model, the response of body neurons in correct trials of our experiment shows a rhythmic baseline shift, a higher response selectivity and reliability, and subsequently a larger difference between the evoked responses to the preferred and non-preferred categories (Figure 9B). On the other hand, in trials lacking the synchronized oscillatory activity, the baseline activity is constantly low. Stimuli presented in this state evoke low-amplitude responses, with less selectivity and reliability, followed by wrong choices (Figure 9C). The response of body neurons in wrong trials of our experiment is consistent with this model (Figure 9D). The model predicts that, for decision making about the stimulus, a decision boundary could be set efficiently in HBT as a result of higher response discriminability and lower response variability. In contrast in LBT, responses to different categories are mixed and no clear decision boundary would exist. Both predictions are consistent with our data.

Various cognitive factors can modulate the baseline activity in the brain. Previous electrophysiological (Chelazzi et al., 1993; Fries et al., 2008) and fMRI (Puri et al., 2009) studies have reported that spatial attention, feature-based attention, and expectation/anticipation of a task-related stimulus can modulate the baseline activity in many cortical areas. This task-related modification of spontaneous neuronal activity is thought to be implemented by top-down control mechanisms (Chawla et al., 1999; Kastner et al., 1999). Some studies have suggested that these top-down effects on the pre-stimulus activity are mediated through an enhanced firing rate (Luck et al., 1997; Super et al., 2003), whereas other studies have reported an enhancement of ongoing theta and alpha oscillations (Busch and VanRullen, 2010; Mo et al., 2011). Consistent but complementary to those findings, our results showed an enhancement of oscillatory spiking activity, in a low-frequency range (<8 Hz), during the preparatory state.

The coupling of synchronized oscillations across different cortices could be a mechanism for the cognitive roles of the neural oscillatory activity. It has been shown that theta-coupling between area V4 and the prefrontal cortex predicts the behavioral performance in a visual memory task (Liebe et al., 2012). It is possible that in our task, the observed oscillatory activity in the IT cortex is coupled with the oscillatory activity in a downstream area such as prefrontal cortex. This coupling could coordinate action potential communication between these areas and facilitate the relay of visual information to the areas that are more involved in conscious perception (Dehaene and Changeux, 2005; Libedinsky and Livingstone, 2011). This hypothesis needs simultaneous recording from IT and prefrontal cortex to be confirmed.

We found a coupling between the ongoing low-frequency oscillation and the spike probability. It has been proposed that specific phases of neural oscillations could create periodic windows of modulated neural excitability (Bishop, 1932; Lee et al., 2005; VanRullen et al., 2005; Haider and McCormick, 2009; Busch and VanRullen, 2010; Haegens et al., 2011). In primary visual cortex, the phase of low-frequency oscillations is realigned by attention, and consequently the attended events occur during the high-excitability phase of the oscillations (Lakatos et al., 2008). Analogously in our experiment, low-frequency (<8 Hz) oscillations were observed before and locked to the stimulus onset (note that long cycles of low-frequency rhythms could cover the 100-ms jitter in the fixation duration). The fact that our effect was observed in the range of delta (<4 Hz) and theta (4–7 Hz) indicates that these two oscillation bands could contribute to similar cognitive functions in the brain. These oscillations provided a precise temporal structure for the modulation of baseline spiking activity and the responses evoked by the stimulus.

The potential impact of rate on the observed power of oscillatory activity might be a concern. However, the relation between firing rate magnitude and power of oscillation has not been completely understood. In fact, for different oscillation frequencies there is no, positive or negative correlation between firing rate and oscillation power. For example, it has been shown that spiking activity is negatively and positively correlated with the power of low and high gamma range, respectively (Ray and Maunsell, 2011). Please note that the large difference observed in the amplitude of oscillation in different frequencies within a very short frequency range (for example, compare the power at 3 and 5 Hz in Figure 3) is not expected merely based on the correlation of oscillation power with firing rate. Additionally, our finding of significant phase locking difference between LBT and HBT does not dependent on the power of the low frequency oscillations in these two conditions. This finding independently validates separation of trials into high and low rate.

We found a cross-frequency coupling between the low-frequency rhythms and the gamma oscillation. The coupling between low- and high-frequency bands has been reported for the ongoing activity in the human brain (Canolty et al., 2006). This coupling coordinates activity in distributed cortical areas providing a more efficient communication among these areas during the cognitive tasks. In our task, the cross-frequency coupling could enhance the neuronal synchronization at the gamma band, which can improve the behavioral performance (Womelsdorf et al., 2006). It has been shown that attention increases the gamma-band synchrony between prefrontal cortex and the visual areas (Gregoriou et al., 2009). Such synchrony might enhance the postsynaptic impact of spikes and improve cross-area communication and neuronal interactions (Womelsdorf et al., 2007).

How could the baseline activity interact with the stimulus-evoked response? At the cellar level, occurrence of few spikes just before the arrival of evoked synaptic activity could increase neural membrane conductance and lead to an enhanced neuronal responsiveness (Haider et al., 2007). At the network level, a rhythmic baseline activity, reflecting a synchronized response of neuron assemblies, could provide a precise temporal window for an efficient integration of synaptic inputs (Volgushev et al., 1998; Schroeder and Lakatos, 2009). This network effect would again increase the likelihood of driving postsynaptic target neurons (Salinas and Sejnowski, 2001; Azouz and Gray, 2003).

The correlation between the baseline activity and detection performance has been reported in other cortices as well (Ress et al., 2000; Super et al., 2003; Addante et al., 2011; Carnevale et al., 2012; Bennett et al., 2013; Spaak et al., 2014). Thus, it is conceivable that the internal state of visual cortex affects sensory processing and behavioral performance in a wide range of cognitive tasks and brain areas. With development of new techniques such as optogenetics, it would be possible to alter the rhythmic background activity of neurons in specific time windows and test the “causal” role of such activity in perception and performance.

Numerous studies have shown that neural oscillation is used to coordinate and synchronize the activity of “population of neurons” with similar or related stimulus-response profile, thereby resulting in perceptual enhancement and improved behavioral performance (Gray et al., 1989; Engel et al., 2001). In trials with high baseline activity, the low-frequency (<8 Hz) rhythms in spiking activity were phase-locked across all trials, cells and sessions. However, this synchrony may not be the only mechanism of signal-to-noise improvement in our task. Here we propose an additional mechanism for the enhancement of neural code and behavioral performance at the level of “single neurons”: if the rhythmic spiking activity happens in a specific time relative to the stimulus onset, it would enhance the responsiveness of single neurons. As a result of such exact timing in all trials and sessions, a synchrony could be also observed at the population level.

Our study provides a single-neuron report on the correlation of the baseline activity in a high-level visual area with the evoked response (its selectivity and variability) and also the behavior. Our findings fill the gap between studies reporting the role of the “brain state” during the pre-stimulus time and studies reporting the role of the “cognitive state” (such as attentional effects) during the evoked response. Furthermore, it is important to note that many electrophysiological studies have largely ignored the role of baseline activity in neural and behavioral effects, mainly by excluding it from the analysis through a “baseline adjustment.” Such a crucial role for the baseline activity could have a profound impact on the way the previous results have been interpreted and also on the future studies.

## Author contributions

Nazli Emadi and Hossein Esteky designed the study. Nazli Emadi collected the data. Nazli Emadi and Reza Rajimehr performed the analysis. Nazli Emadi, Reza Rajimehr and Hossein Esteky wrote the manuscript.

### Conflict of interest statement

The authors declare that the research was conducted in the absence of any commercial or financial relationships that could be construed as a potential conflict of interest.
